# Molecular Insights into Short QT Syndrome

**DOI:** 10.19102/icrm.2018.090302

**Published:** 2018-03-15

**Authors:** Srikanth Perike, Mark D. McCauley

**Affiliations:** ^1^Department of Medicine, Section of Cardiology, Department of Bioengineering, Department of Physiology and Biophysics, College of Medicine, University of Illinois at Chicago, Chicago, IL, USA

**Keywords:** Atrial arrhythmia, genetic arrhythmia, pharmacotherapy, short QT syndrome, ventricular arrhythmia

## Abstract

Short QT syndrome (SQTS) is a myocardial conduction disorder characterized by a short QT interval on electrocardiogram and predisposition to familial atrial fibrillation and/or sudden cardiac death. Genetic SQTS is primarily caused by one or more cardiac ion channelopathies, in which either impaired depolarization currents, or enhanced repolarization currents, shorten cardiac action potential duration. Given that QT interval duration is not always predictive of arrhythmia burden and risk of death in SQTS, there is a need to understand the molecular mechanisms of the condition to improve risk prognostication and potential pharmacologic treatment. In the last decade, several computational advances and in vitro** preclinical studies have provided insight into the molecular mechanisms underlying congenital SQTS. In this review, we discuss recent findings in SQTS molecular mechanisms and correlate these advances with clinical guidelines for SQTS diagnosis and treatment.

## Introduction

Short QT syndrome (SQTS) is a genetic cardiac channelopathy with a predisposition to life-threatening atrial and ventricular arrhythmias. Clinically, SQTS is associated with a variety of signs and symptoms including palpitations, presyncope, syncope, shortened atrial and ventricular effective refractory periods, and sudden cardiac death.^1^ The term “idiopathic SQTS” was first used by Gussak et al. in 2000 to describe a new clinical syndrome.^2^ Despite patients presenting with normal cardiac anatomy, SQTS is characterized on electrocardiogram (ECG) by short QT intervals, representing cardiac depolarization and repolarization, and ranging from as low as 260 ms to 280 ms, to approximately 360 ms.^3^ ECG findings revealed that SQTS is associated with accelerated cardiac repolarization, which causes tall, symmetrical, or asymmetrical peaked T-wave morphology and absence of an ST segment.^4^ The arrhythmogenic potential and high incidence of familial nature of SQTS was reported by Gaita et al. in 2003.^5^ The genetic screening of affected individuals demonstrates that SQTS pathogenesis is associated with abnormalities in cardiac ion channels that regulate cardiac action potentials. For instance, gain-of-function genetic mutations in voltage-gated potassium channel subunits *KCNH2*,^6^ *KCNQ1*,^7^ and *KCNJ2*^7^ are known to cause SQTS; conversely, loss-of-function mutations in cardiac voltage-gated calcium channels including *CACNA1C*,^8^ *CACNB2B*,^8^ and *CACNA2D*^9^ cause SQTS and have phenotypic overlap with Brugada syndrome.^8,9^

Aside from genetic channelopathies, other acquired causes of short QT interval must be considered before entertaining the diagnosis of genetic SQTS. For example, hypercalcemia,^10^ hyperkalemia,^11^ acidosis,^11^ vagal or sympathetic hypertonia,^11^ effects of digitalis,^12^ and androgen misuse^13^ are all associated with QT interval shortening. In this review, we will discuss recent advances in understanding the consequences of genetic SQTS, pharmacotherapy, and diagnostic challenges. Given the relative paucity of genetic SQTS—fewer than 200 SQTS patients have been identified worldwide—preclinical models of cellular SQTS mechanisms are necessary for timely diagnosis and treatment of this potentially lethal disease.

## Cellular and tissue models of short QT syndrome

There is a lack of accurate cellular and tissue models of the human ventricles that recapitulate abnormal ion channel kinetics in SQTS. To overcome this limitation, several groups have established *in silico* modeling to determine the molecular underpinnings of QT interval shortening. The first mathematical model for human ventricular myocytes was established by Priebe and Beuckelmann in 1998.^14^ This model was derived via parameter variations from the Luo-Rudy phase II model (LR II model) of guinea pig ventricular cells.^15^ Priebe and Beuckelmann obtained experimental data for major ionic currents from human myocytes including *I*_Kr_, *I*_Ks_, *I*_Ca_, *I*_to_, and *I*_K1_, and adopted *I*_Na_, *I*_NaCa_, and *I*_NaK_ currents from the LR II model scaled to fit human ventricular cell data. Bernus et al. simplified the Priebe and Beuckelmann model by fixing the intracellular ionic concentrations and reformulating the currents that can be applied to any ionic model.^16^ This model is computationally efficient, less complex versus the second-generation Priebe and Beuckelmann ionic model, and more accurate for studying reentrant phenomena in human ventricular tissue.^16^ In 2004, ten Tusscher et al. developed a ventricular model that reproduced the detailed electrophysiological properties of single human ventricular cells for studying reentrant arrhythmias and performing large-scale spatial simulations.^17^ They have used new formulations for ionic currents and combined experimental data from human ventricular cells and ion channel expression experiments. While each of these approaches has been instrumental in understanding the mechanisms of ventricular arrhythmias, none of them incorporated the effects of specific genetic mutations. Thus, to better understand the molecular and cellular bases of specific mutations, experimental observations of ion channel-specific disease-causing mutations were included in subsequent *in silico* analyses.

*In silico* models have replicated experimental data describing the loss of *I*_Kr _inactivation, which leads to a gain in *I*_Kr_ function and shortens the QT interval in this syndrome.^18^ Priori et al. demonstrated that in the setting of SQTS 3 (SQT3), a gain-of-function mutation (D172N) in the *KCNJ2* gene, encoding the inwardly rectifying *K*_IR_ 2.1 (I_K1_) channel, is associated with an accelerated repolarization with a predisposition to ventricular reentry.^19^ Adeniran et al. reproduced this in SQTS 1 (SQT1)^20^ and SQT3^21^ by simulating the data for wild-type and mutant versions of the *KCNH2* and *KCNJ2* genes. The group found that an N588K-*hERG* mutation in SQT1 initiates and maintains ventricular reentry, which increases the generation of reentrant spiral waves and stabilizes scroll waves in anatomical three-dimensional (3D) ventricular tissue.^20^ On the other hand, in SQT3, the gain-of-function *K*_IR _2.1 D172N mutation increases *I*_K1_ ionic currents and increases arrhythmia risk due to enhanced tissue vulnerability, a reduced ventricular effective refractory period, and altered membrane excitability.^21^ Rice et al. developed a related myocyte contraction model primarily based on a cross-bridge cycling model of cardiac muscle contraction.^22^ This model simulates a wide variety of experimental cardiac muscle characteristics such as steady-state force-sarcomere length, force-calcium, and sarcomere length-calcium relations.^22^ At present, there is little information available regarding impaired cardiac contractile functions in SQTS patients. To address this limitation, Adeniran et al. coupled cardiac muscle contraction and ventricular cell models; SQTS mutations were simulated at the single-cell level and evaluated with respect to the potential electromechanical consequences.^23^ They also built experimental cellular data into predictions for mechanical activity in two-dimensional and 3D human ventricular tissue models, and these geometrical changes were incorporated into the electrophysiological computations.^24^ They found that shortening of the action potential in SQTS is associated with reduced ventricular mechanical function. Collectively, *in silico* modeling of the human heart has provided powerful insights into the mechanisms of arrhythmias and genetic consequences of physicomechanical interactions in SQTS.

## Short QT syndrome in experimental models

The mechanism of arrhythmogenesis in SQTS is less clear, and few SQTS cohorts have been identified. At present, electrophysiologic testing and genetic screening of patients and their families are performed to improve risk stratification for affected kindred. Several groups have established a link between preclinical models and a pharmacogenetic approach to treating patients with SQTS. For instance, a gain-of-function (T618I) mutation in the *hERG* K^+^ channel protein was identified in families with heritable SQTS. El Harchi et al. electrophysiologically characterized the T618I mutation, which exerts a more modest effect on cardiac action potential repolarization compared with other *I*_hERG_ variants of SQT1.^25^ Additionally, the group was able to inhibit *I*_hERG_-T618I in SQT1 using quinidine, disopyramide, D-sotalol, and flecainide.^25^ Antzelevitch et al. genetically screened 82 probands with Brugada syndrome for ion channel abnormalities.^8^ They performed site-directed mutagenesis experiments for wild-type and mutant *CACNB2B*, *CACNA2D1*, and *CACNA1C* channels and electrophysiologically characterized the mutations in mammalian cells. They found that loss-of-function mutations in α_1_- and β-subunits encoding the cardiac L-type calcium channel are associated with QT interval shortening.^8^ Tse et al. characterized the ventricular arrhythmogenic properties of hyperkalemia in Langendorff-perfused mouse hearts.^26^ They found that hyperkalemia exerted proarrhythmic effects by shortening the effective refractory period and action potential duration, which leads to a decrease in the excitation wavelength and predisposes to reentry of ventricular tachyarrhythmias.^26^ In contrast, hypercalcemia exerted antiarrhythmic effects by changing the ventricular effective refractory period, which is in accordance with the ventricular effective refractory period/latency ratio and critical intervals.^26^

Given that patients with SQTS are prone to the development of polymorphic ventricular tachycardia, including *torsades de pointes*, Extramiana and Antzelevitch developed a canine left ventricular wedge model that showed a heterogeneous distribution of action potentials in the left ventricular wall, increasing the transmural dispersion of repolarization.^27^ The group found that transmural dispersion is critical for inducing polymorphic ventricular tachycardia and is a significant trigger for SQTS-related arrhythmias.^27^ Further work by the group in 2008 evaluated a left ventricular wedge model that recapitulates electrocardiographic and arrhythmic manifestations of SQT1.^28^ The inclusion of potassium channel “openers” increases the transmural dispersion repolarization and reduces the ventricular effective refractory period.^28^ Evidence is also accumulating that patient-specific induced pluripotent stem cells could be used to test the potential of antiarrhythmic agents to prolong the QT interval.^29–31^ This technology could directly model rare forms of SQTS in patients and kindred for whom a pharmacogenetics approach is unclear. Experimental models have been critical to understanding SQTS pathogenesis and potential therapies.

## Diagnosis of short QT syndrome in human patients

The number of identified congenital SQTS cohorts is small, and establishing the diagnosis is usually quite challenging. Additionally, there is no proper consensus on the exact cutoff value for shorter corrected QT intervals that could confer a higher risk of cardiac arrhythmia.^1,32,33^ In 2011, Gollob et al. proposed four diagnostic criteria for SQTS, focusing on ECG findings of shortened QT interval, clinical history, family history, and genotyping.^3^ Initially, genetic screening and identification of the short QT interval on ECG would be helpful for affected individuals. Certainly, this will enable identification of the abnormal genetic variants that can be targeted for the optimal diagnosis and could help reduce the prevalence in at-risk family members. The European Society of Cardiology recently developed guidelines on SQTS diagnosis **(Table 1)**.^34^

## Short QT syndrome pharmacotherapy

To date, an implantable cardioverter-defibrillator (ICD) is the first-line therapy for patients with symptomatic SQTS.^5,35,36^ Patients who have survived a previous cardiac arrest and/or those with documented occurrence of spontaneous sustained ventricular tachyarrhythmias with or without syncope have been suggested as candidates to receive ICDs for secondary prevention of cardiac arrest (class I recommendation) **(Table 2)**.^34^ Since ICD therapy has been implemented for high-risk patients, the recurrence rate of cardiac arrest has been estimated at 10% per year.^32^ However, an ICD implant may not be feasible in all patients, especially in those who are very young (infants) SQTS patients with severe phenotypes. Pharmacotherapy is a primary adjunctive modality to ICD to reduce arrhythmia burden and reduce potential ICD shocks. However, antiarrhythmic drug use requires a comprehensive risk stratification and management plan. Asymptomatic patients also require risk stratification, in which ICD implantation and/or pharmacotherapy is recommended. QT prolongation can be achieved by pharmacological agents that reduce outward potassium currents and/or enhance the inward flow of current. Gaita et al. tested four different antiarrhythmic agents (flecainide, sotalol, ibutilide, and hydroquinidine) to prolong the QT interval and prevent arrhythmia recurrence in SQT1 patients.^37^ More specifically, hydroquinidine prolonged QT intervals to near-normal levels, from 263 ± 12 ms to 362 ± 25 ms, and was associated with an increase in ventricular effective refractory period, as well as the prevention of ventricular fibrillation.^37^ Notably, hydroquinidine-treated patients were followed for a year and remained asymptomatic without any episodes of ventricular arrhythmia. This suggests that hydroquinidine is the most effective antiarrhythmic agent for SQTS patients to date. Milberg et al. demonstrated that the antiarrhythmic effects of quinidine in SQTS are due to less repolarization dispersion and prolonged post-repolarization refractoriness.^38^ Another antiarrhythmic agent, flecainide, slightly prolonged the QT interval primarily due to prolongation of the QRS complex, which may be the second choice for patients who are intolerant to quinidine therapy.^37^ In contrast, sotalol and ibutilide have no effect on QT interval in this population.^37^ Propafenone prevents paroxysms of atrial fibrillation without arrhythmia recurrence in SQTS patients.^36^ The Vaughn-Williams class III antiarrhythmic drug nifekalant, a pure *I*_Kr_ blocker, is effective in treating ventricular tachycardia storm by prolonging atrial and ventricular effective refractory periods and normalizing the QT interval.^39,40^ In SQT1, disopyramide (a class Ia antiarrhythmic against the *hERG* channel) is effective in that it prolongs the QT interval and ventricular refractory period and abbreviates the T_peak_ to T_end_ interval.^41^ Isoproterenol infusion has been effectively used to manage electrical storm in SQTS.^42^ Existing pharmacotherapy options for SQTS patients are still poorly defined due to the small number of affected cohorts, but, based on the accumulating evidence and guidelines, hydroquinidine may be the ideal choice.

## Utility of computational and experimental findings for further diagnosis and treatment

To improve pharmacologic options for SQTS, we require a better understanding of arrhythmia mechanisms, which will allow us to tailor rational drug designs to specific underlying ion channelopathies. Computational models have been successfully utilized to assess effects on drug–ion channel interactions and screen the arrhythmogenic potential of pharmacological agents. Luo et al. utilized computational modeling to assess the antiarrhythmic effects of amiodarone on human ventricular electrophysiology in SQT3.^43^ At the single-cell level, they found that amiodarone use increased action potential duration, the effective refractory period, and the QT interval. This suggests that amiodarone may be a potential pharmacological agent for preventing arrhythmogenesis in SQT3 patients. The same group assessed the antiarrhythmogenic effects of E-4031, disopyramide, and quinidine on SQT1 using a mathematical model of human ventricular electrophysiology.^44^ They found that E-4031 and disopyramide exerted no significant effects on ventricular cell action potential duration, even at 90% repolarization, as compared with quinidine. Additionally, quinidine prolonged the QT interval by decreasing the T-wave amplitude, increasing the effective refractory period, and consequently terminating the reentrant ventricular arrhythmias in SQTS.^44^ They also simulated the pharmacological actions of quinidine that exhibited antiarrhythmic effects in SQT1. Their results revealed a causal link between quinidine and QT interval prolongation in SQT1, indicating that it may be a first-line antiarrhythmic agent for SQT1. Ji et al. developed an experimental compound, PA-6, which is a new drug candidate for targeting the inward rectifier potassium current *I*_K1_.^45^ They found that the *KCNJ2* gain-of-function mutations V93I and D172N in the *K*_IR_ 2.1 ion channel do not impair PA6-mediated inhibition of I_K1_. Additionally, PA6 enhances *K*_IR_ 2.1 ion channel protein expression and intracellular accumulation, making it a drug candidate for SQT3. The clinical application of insights from molecular modeling and preclinical results could effectively translate information for the benefit of SQTS and congenital atrial fibrillation patients.

## Conclusions

SQTS is an inherited ion channelopathy characterized by high incidences of life-threatening atrial and ventricular arrhythmias and sudden cardiac death. The small number of cohorts and the overlapping range of ECG values between cohorts and healthy individuals sometimes make this syndrome difficult to diagnose. Despite the challenges, however, with the emergence of *in silico* investigations and preclinical electrophysiological studies, we are better able to predict possible candidate drugs to treat genetic SQTS. That being said, a pharmacogenomic approach to SQTS is still in its infancy, and more studies are required. This review highlighted how the genetic characterization of cohorts with SQTS allows for the prediction of arrhythmia mechanisms and drug–gene interactions. Diagnosis remains challenging and is primarily based on ECG measurements of the QT interval, clinical and familial history, and genetic findings. Currently, the ICD is the first-choice prophylactic therapy for symptomatic SQTS patients at risk for sudden arrhythmic death. In recent years, various antiarrhythmic agents have been tested to prolong QT intervals, including flecainide, sotalol, ibutilide, and hydroquinidine. Numerous studies have indicated that hydroquinidine is the most promising antiarrhythmic agent. Future research should also focus on other factors such as electrolyte imbalances, epigenetics, and competitive physical activity in SQTS patients. This will help facilitate more timely diagnosis and is crucial to prevent the recurrence of life-threatening arrhythmias.

## Figures and Tables

**Table 1:** ESC Recommendations for SQTS Diagnosis^34^

	Class of Recommendation	Level of Evidence
SQTS is diagnosed in the presence of a QTc ≤ 340 ms	I	C
SQTS should be considered in the presence of a QTc ≤360 ms and one or more of the following: 1. a confirmed pathogenic mutation; 2. a family history of SQTS; 3. a family history of sudden death < 40 years of age; and/or 4. survival following an episode of ventricular tachycardia/ventricular fibrillation in the absence of heart disease.	IIa	C

SQTS: short QT syndrome; QTc: corrected QT interval.

**Table 2:** ESC Recommendations for Risk Stratification and Management in SQTS^34^

ESC 2015 SQTS Recommendations	Class of Recommendation	Level of Evidence	Other Studies Supporting Recommendations
ICD implantation is recommended in patients with a diagnosis of SQTS who: 1. are survivors of an aborted episode of cardiac arrest; and/or 2. have documented spontaneous sustained ventricular tachycardia.	I	C	• Gaita et al.^5^ • Goldenberg et al.^35^
Quinidine or sotalol may be considered in patients with a diagnosis of SQTS who qualify for an ICD but who present a contraindication to the ICD implant, or who refuse to undergo implantation.	IIb	C	• Gaita et al.^37^ • Luo et al.^43^
Quinidine or sotalol may be considered in asymptomatic patients with a diagnosis of SQTS and a family history of sudden cardiac death.	IIb	C	• Gaita et al.^37^ • Luo et al.^43^
Invasive electrophysiological study with programmed ventricular stimulation for sudden cardiac death risk stratification.	III	C	• Mazzanti et al.^32^ • Luo et al.^43^

ESC: European Society of Cardiology; SQTS: short QT syndrome; ICD: implantable cardioverter-defibrillator.

## References

[1] Giustetto C, Di Monte F, Wolpert C (2006). Short QT syndrome: clinical findings and diagnostic-therapeutic implications. Eur Heart J..

[2] Gussak I, Brugada P, Brugada J (2000). Idiopathic short QT interval: a new clinical syndrome?. Cardiology.

[3] Gollob MH, Redpath CJ, Roberts JD (2011). The short QT syndrome: proposed diagnostic criteria. J Am Coll Cardiol..

[4] Patel C, Yan GX, Antzelevitch C (2010). Short QT syndrome: from bench to bedside. Circ Arrhythm Electrophysiol..

[5] Gaita F, Giustetto C, Bianchi F (2003). Short QT syndrome: a familial cause of sudden death. Circulation..

[6] Brugada R, Hong K, Dumaine R (2004). Sudden death associated with short-QT syndrome linked to mutations in HERG. Circulation..

[7] Bellocq C, van Ginneken AC, Bezzina CR (2004). Mutation in the KCNQ1 gene leading to the short QT-interval syndrome. Circulation..

[8] Antzelevitch C, Pollevick GD, Cordeiro JM (2007). Loss-of-function mutations in the cardiac calcium channel underlie a new clinical entity characterized by ST-segment elevation, short QT intervals, and sudden cardiac death. Circulation..

[9] Templin C, Ghadri JR, Rougier JS (2011). Identification of a novel loss-of-function calcium channel gene mutation in short QT syndrome (SQTS6). Eur Heart J..

[10] Bjerregaard P, Nallapaneni H, Gussak I (2010). Short QT interval in clinical practice. J Electrocardiol..

[11] Maury P, Extramiana F, Sbragia P (2008). Short QT syndrome. Update on a recent entity. Arch Cardiovasc Dis..

[12] Cheng TO (2004). Digitalis administration: an underappreciated but common cause of short QT interval. Circulation..

[13] Hancox JC, Choisy SC, James AF (2009). Short QT interval linked to androgen misuse: wider significance and possible basis. Ann Noninvasive Electrocardiol..

[14] Priebe L, Beuckelmann DJ (1998). Simulation study of cellular electric properties in heart failure. Circ Res..

[15] Luo CH, Rudy Y (1994). A dynamic model of the cardiac ventricular action potential. II. Afterdepolarizations, triggered activity, and potentiation. Circ Res..

[16] Bernus O, Wilders R, Zemlin CW, Verschelde H, Panfilov AV (2002). A computationally efficient electrophysiological model of human ventricular cells. Am J Physiol Heart Circ Physiol..

[17] ten Tusscher KH, Noble D, Noble PJ, Panfilov AV (2004). A model for human ventricular tissue. Am J Physiol Heart Circ Physiol..

[18] Zhang H, Hancox JC (2004). In silico study of action potential and QT interval shortening due to loss of inactivation of the cardiac rapid delayed rectifier potassium current. Biochem Biophys Res Commun..

[19] Priori SG, Pandit SV, Rivolta I (2005). A novel form of short QT syndrome (SQT3) is caused by a mutation in the KCNJ2 gene. Circ Res..

[20] Adeniran I, McPate MJ, Witchel HJ, Hancox JC, Zhang H (2011). Increased vulnerability of human ventricle to re-entrant excitation in hERG-linked variant 1 short QT syndrome. PLoS Comput Biol..

[21] Adeniran I, El Harchi A, Hancox JC, Zhang H (2012). Proarrhythmia in KCNJ2-linked short QT syndrome: insights from modelling. Cardiovasc Res..

[22] Rice JJ, Wang F, Bers DM, de Tombe PP (2008). Approximate model of cooperative activation and crossbridge cycling in cardiac muscle using ordinary differential equations.. Biophys J.

[23] Adeniran I, Hancox JC, Zhang H (2013). In silico investigation of the short QT syndrome, using human ventricle models incorporating electromechanical coupling. Front Physiol..

[24] Pathmanathan P, Whiteley JP (2009). A numerical method for cardiac mechanoelectric simulations. Ann Biomed Eng..

[25] El Harchi A, Melgari D, Zhang YH, Zhang H, Hancox JC (2012). Action potential clamp and pharmacology of the variant 1 Short QT Syndrome T618I hERG K(+) channel. PLoS One..

[26] Tse G, Sun B, Wong ST, Tse V, Yeo JM (2016). Anti-arrhythmic effects of hypercalcemia in hyperkalemic, Langendorff-perfused mouse hearts. Biomed Rep..

[27] Extramiana F, Antzelevitch C (2004). Amplified transmural dispersion of repolarization as the basis for arrhythmogenesis in a canine ventricular-wedge model of short-QT syndrome. Circulation..

[28] Patel C, Antzelevitch C (2008). Cellular basis for arrhythmogenesis in an experimental model of the SQT1 form of the short QT syndrome. Heart Rhythm..

[29] Itzhaki I, Maizels L, Huber I (2011). Modelling the long QT syndrome with induced pluripotent stem cells. Nature..

[30] Matsa E, Rajamohan D, Dick E (2011). Drug evaluation in cardiomyocytes derived from human induced pluripotent stem cells carrying a long QT syndrome type 2 mutation. Eur Heart J..

[31] Moretti A, Bellin M, Welling A (2010). Patient-specific induced pluripotent stem-cell models for long-QT syndrome. N Engl J Med..

[32] Mazzanti A, Kanthan A, Monteforte N (2014). Novel insight into the natural history of short QT syndrome. J Am Coll Cardiol..

[33] Rudic B, Schimpf R, Borggrefe M (2014). Short QT syndrome – review of diagnosis and treatment. Arrhythm Electrophysiol Rev..

[34] Priori SG, Blomstrom-Lundqvist C, Mazzanti A (2015). 2015 ESC Guidelines for the management of patients with ventricular arrhythmias and the prevention of sudden cardiac death: The Task Force for the Management of Patients with Ventricular Arrhythmias and the Prevention of Sudden Cardiac Death of the European Society of Cardiology (ESC). Endorsed by: Association for European Paediatric and Congenital Cardiology (AEPC). Eur Heart J..

[35] Goldenberg I, Gillespie J, Moss AJ (2010). Long-term benefit of primary prevention with an implantable cardioverter-defibrillator: an extended 8-year follow-up study of the Multicenter Automatic Defibrillator Implantation Trial II. Circulation..

[36] Bjerregaard P, Gussak I (2005). Short QT syndrome. Ann Noninvasive Electrocardiol..

[37] Gaita F, Giustetto C, Bianchi F (2004). Short QT syndrome: pharmacological treatment. J Am Coll Cardiol..

[38] Milberg P, Hilker E, Ramtin S (2007). Proarrhythmia as a class effect of quinolones: increased dispersion of repolarization and triangulation of action potential predict torsades de pointes. J Cardiovasc Electrophysiol..

[39] Mizobuchi M, Enjoji Y, Yamamoto R (2008). Nifekalant and disopyramide in a patient with short QT syndrome: evaluation of pharmacological effects and electrophysiological properties. Pacing Clin Electrophysiol..

[40] Chinushi M, Sato A, Izumi D, Furushima H (2012). Nifekalant enlarged the transmural activation-recovery interval difference as well as the peak-to-end interval on surface ECG in a patient with short-QT syndrome. J Cardiovasc Electrophysiol..

[41] Schimpf R, Veltmann C, Giustetto C, Gaita F, Borggrefe M, Wolpert C (2007). In vivo effects of mutant HERG K+ channel inhibition by disopyramide in patients with a short QT-1 syndrome: a pilot study. J Cardiovasc Electrophysiol..

[42] Bun SS, Maury P, Giustetto C, Deharo JC (2012). Electrical storm in short-QT syndrome successfully treated with Isoproterenol. J Cardiovasc Electrophysiol..

[43] Luo C, Wang K, Zhang H (2017). Effects of amiodarone on short QT syndrome variant 3 in human ventricles: a simulation study. Biomed Eng Online..

[44] Luo C, Wang K, Zhang H (2017). In silico assessment of the effects of quinidine, disopyramide and E-4031 on short QT syndrome variant 1 in the human ventricles. PLoS One..

[45] Ji Y, Veldhuis MG, Zandvoort J (2017). PA-6 inhibits inward rectifier currents carried by V93I and D172N gain-of-function KIR2.1 channels, but increases channel protein expression. J Biomed Sci..

